# Case report: Two-step lamellar keratoplasty for alkali burns

**DOI:** 10.3389/fmed.2024.1496083

**Published:** 2024-10-21

**Authors:** Xie Fang, Zhiwen Xie, Shunrong Luo, Xianwen Xiao, Zhengwei Yang, Yuan Lin, Huping Wu

**Affiliations:** ^1^Xiamen Eye Center and Eye Institute of Xiamen University, School of Medicine, Xiamen, China; ^2^Xiamen Clinical Research Center for Eye Diseases, Xiamen, China; ^3^Xiamen Key Laboratory of Ophthalmology, Xiamen, China; ^4^Fujian Key Laboratory of Corneal and Ocular Surface Diseases, Xiamen, China; ^5^Xiamen Key Laboratory of Corneal and Ocular Surface Diseases, Xiamen, China; ^6^Translational Medicine Institute of Xiamen Eye Center of Xiamen University, Xiamen, China

**Keywords:** alkali burns, ocular surface, lamellar keratoplasty, corneal perforation, two-step lamellar keratoplasty, femtosecond laser

## Abstract

**Purpose:**

To report a case of a patient with ocular surface alkali burn who developed corneal perforation before entering a stable phase. This patient was treated with a custom-designed lamellar graft using a Two-Step lamellar keratoplasty (LK) after 3 months alkali burn.

**Methods:**

This study was a case report.

**Results:**

A 43-year-old male patient who presented with redness, pain, and decreased vision in his left eye following lime exposure. Initial treatment involved amniotic membrane transplantation and anterior chamber irrigation. However, the patient subsequently developed corneal infiltration and progressive thinning. The patient refused the corneal transplantation and ultimately underwent tarsorrhaphy. Twenty days postoperatively, the patient experienced sudden ocular pain and central corneal perforation. Considering the potential complications of conventional corneal transplantation, including stem cell deficiency, graft dissolution, and rejection, a personalized Two-Step LK was devised. Following this procedure, the patient’s vision gradually improved to 20/133 (without correction), with a good corneal condition but mild epithelial defects and edema. At the 8-month follow-up, subepithelial corneal haze was observed, but uncorrected visual acuity remained stable at 20/133 and best corrected visual acuity was up to 20/66.

**Conclusion:**

For patients with ocular surface alkali burns who experience persistent disease progression despite early and mid-stage aggressive interventions, this study is the first to report on the use of a Two-Step LK. This approach takes into account both the “soil” factors that may lead to graft dissolution and the “seed” factors related to recipient stem cell deficiency. The results in preventing graft dissolution and maintaining postoperative corneal function are encouraging.

## Introduction

Ocular alkali burns are the common and challenging type to treat. Severe alkali burns can cause extensive structural damage and deep tissue penetration, leading to significant corneal dysfunction and disruption of ocular surface homeostasis. This imbalance can result in the rapid progression of the disease (1–3). We report a case of a patient with ocular surface alkali burn who developed corneal perforation before reaching the stable phase. Considering the patient was at high risk for graft dissolution following corneal transplantation. Therefore, we designed a two-step lamellar keratoplasty (LK) to reconstruct the ocular surface. This approach ensured postoperative graft stability and facilitated functional recovery.

## Report of case

**Figure 1:**
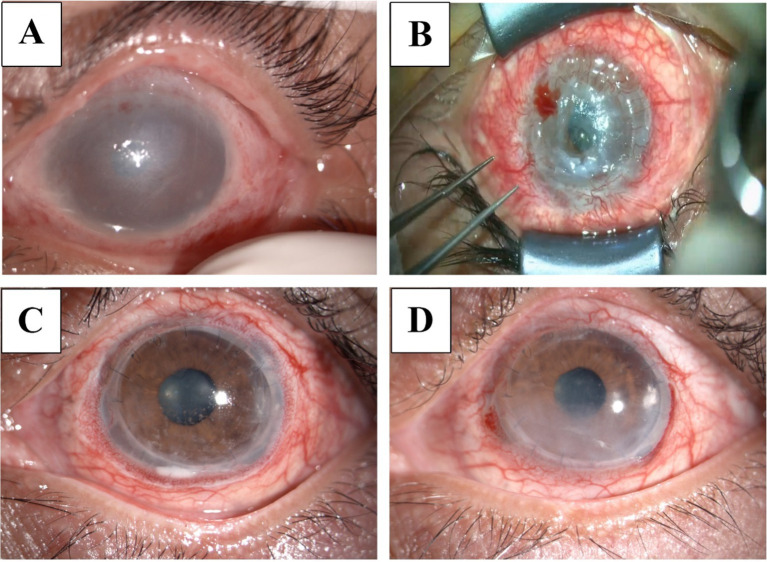
Slit-lamp examination of the patient. (A) Slit lamp photo of the left eye post-injury, showing significant edema and cloudy infiltration. (B) Twenty days after eyelid suturing, the surgical microscope revealed a 6 mm diameter central corneal perforation with iris prolapse. (C) Two months post-surgery, the graft remained clear with scattered epithelial defects. (D) Eight months post-surgery, the graft showed cloudiness beneath the cornea and a few peripheral neovascularizations.

A 43-year-old male, with redness and pain in the left eye accompanied by a sudden decrease in vision after exposure to lime for 1 day. The patient has accepted our treatment for a day after injury. His uncorrected visual acuity (UCVA) was 20/1000 OS, which cannot be corrected, and intraocular pressure (IOP) was 11.9 mm Hg OS. Slit lamp examination revealed significant edema (Figure 1A). Treatment included amniotic membrane transplantation in the left eye combined with anterior chamber irrigation. The postoperative treatment regimen consisted of topical application of deproteinized calf serum eye drops, hydroxypropyl methylcellulose eye drops, and loteprednol-tobramycin eye drops, along with systemic intravenous administration of 80 mg of methylprednisolone.

**Figure 2:**
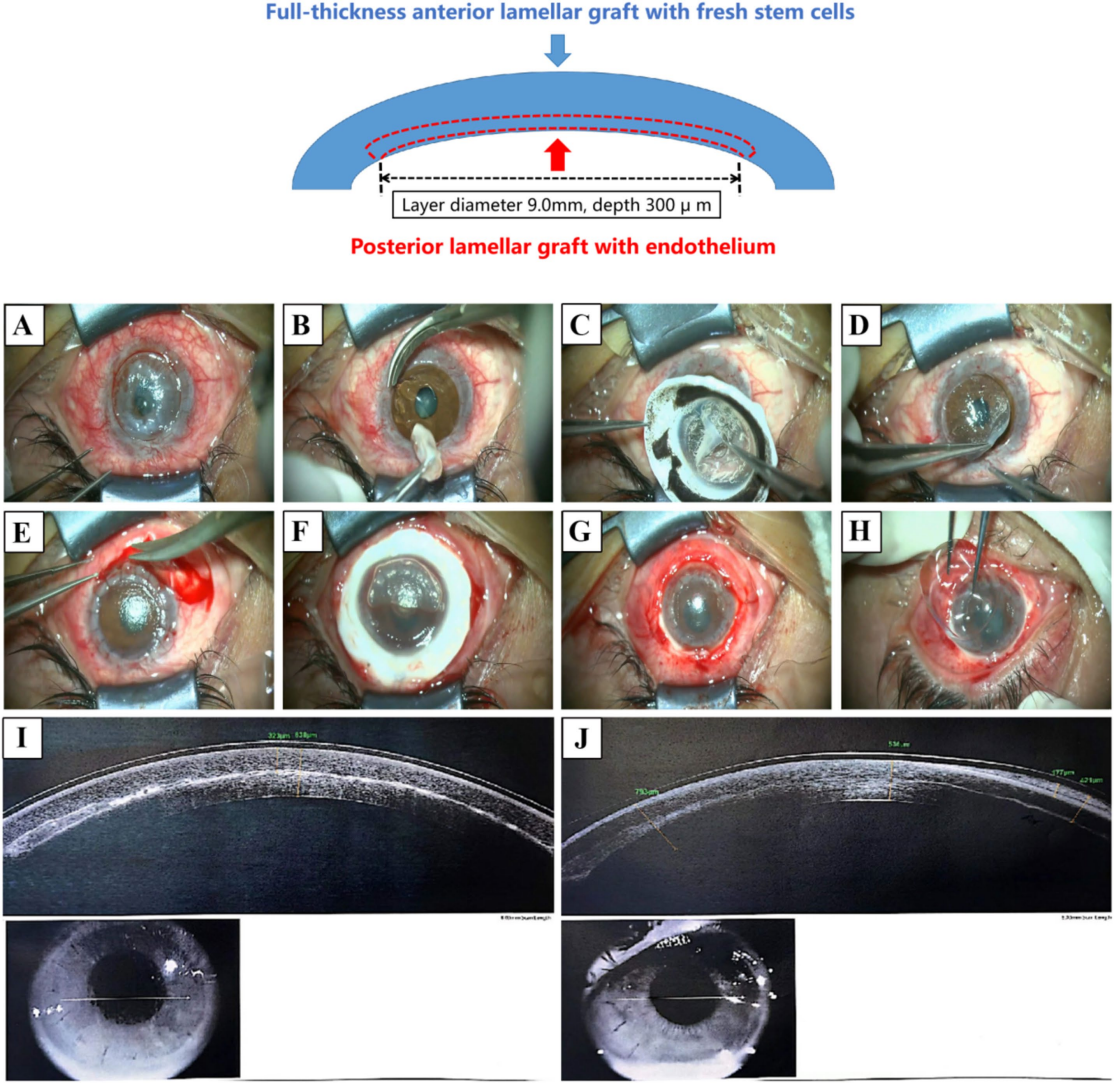
Two-step lamellar transplantation surgery. Using femtosecond laser-assisted technology, the graft from the same donor was divided into a posterior lamellar graft with endothelial cells and an anterior lamellar graft with fresh limbus. The transplantation was performed in two steps. (A,B) A trephine (diameter 8.5 mm) and corneal scissors are used to prepare the recipient bed and treat the lesion. (C,D) In the first step, the posterior lamellar graft was transplanted and sutured. (E–H) The surrounding neovascularization and old scar tissue were removed. In the second step, the anterior lamellar graft was transplanted to cover the area, and the surrounding conjunctiva was reattached before applying a bandage lens. (I) AS-OCT scan 3 days postoperatively. (J) AS-OCT scan 2 months postoperatively.

Two months later, the patient exhibited progressive thinning with infiltration beneath the cornea near the margin of the left eye. Anterior segment optical coherence tomography (AS-OCT) measured a corneal thickness of approximately 355 μm, and visual acuity decreased to finger counting OS with an IOP of 13 mm Hg OS. The patient was advised to undergo tarsorrhaphy, which he refused, opting instead for a second amniotic graft. Seven days post-surgery, the entire cornea showed gradual thinning, with AS-OCT measuring a central corneal thickness of about 254 μm and the thinnest area at 203 μm. Corneal transplantation was recommended, but the patient chose to undergo tarsorrhaphy in the end. Twenty days after the tarsorrhaphy, there was significant pain and central corneal perforation (Figure 1B). At this point, 3 months have passed since the alkali burn, and the patient ultimately consented to undergo corneal transplantation. Considering the potential complications such as stem cell deficiency, graft dissolution, and rejection associated with penetrating keratoplasty (PK), the choice between Deep anterior LK also entails the risk of poor graft-host bed attachment. A personalized two-step LK was designed (Figure 2).

Postoperatively, BCVA improved to 20/1000 OS after 3 days, 20/400 after 10 days, and 20/133 at 2 months (Figure 1C). AS-OCT images taken post-surgery showed good attachment between the anterior and posterior lamellar implants (Figures 2I,J). The postoperative follow-up lasted for 8 months. During this period, the patient reported mild foreign body sensation and dryness, significantly alleviated with appropriate eye drops and eyelid gland physical therapy. At the eighth-month postoperative visit, slit lamp examination showed that UCVA was at 20/133 OS, and BCVA was 20/66 (Figure 1D).

## Discussion

The surgical approach for severe ocular surface burns aims to reconstruct ocular surface function, address eyelid deformities, correct limbal stem cell deficiency, and alleviate conjunctival sac constriction, preparing the eye for future vision restoration surgeries (4). Patients with severe corneal burns, especially those caused by alkali, often experience serious complications before reaching a stable period. The condition is complex, progresses rapidly, and is challenging to treat (5). The persistent inflammatory response, repair disorders, and ongoing structural loss of the primary cornea complicate treatment and surgical options (6). In this case, considering the high-risk nature of postoperative patients, PK often results in stem cell deficiency, graft melting, and rejection. Meanwhile, full-thickness lamellar keratoplasty may fail due to poor graft-to-recipient bed adhesion. Therefore, designing a personalized transplantation strategy to address these challenges effectively is crucial.

In this case, we employed a two-step LK to remove the “soil” factors that could lead to graft dissolution and address the “seed” factor of recipient stem cell deficiency. The implementation of the two-step LK involves using the same donor graft to prepare a posterior lamellar graft for the first-step transplantation while preserving the anterior lamellar graft with fresh stem cells for the second-step transplantation. First, under the assistance of an artificial anterior chamber system, femtosecond laser cutting parameters are set according to the posterior cut diameter required for the graft. The corneal diameter and depth are adjusted for posterior lamellar cutting, including the depth of the anterior surface, with a corneal flap edge angle of 90 degrees. The femtosecond laser energy is set to 2.0 (as shown in Figure 2 for the cutting depth and diameter in this case). Once the graft preparation is complete, the posterior lamellar graft is sutured over the recipient bed (Figures 2C–E). Finally, the anterior lamellar graft is used for ocular surface reconstruction (Figures 2F–H).

Therefore, surgery for alkali burns must consider both visual function recovery and ocular surface conditions. The potential long-term complications following the two-step LK procedure in this case were a key area of concern, such as corneal haze, neovascularization, or recurrence of epithelial defects. During follow-up, the patient experienced mild irritation with symptoms of dry eyes and foreign body sensation. One year later, localized corneal haze was observed, which may be attributed to corneal exposure caused by eyelid margin damage from the alkali burn. Therefore, determining the optimal timing for postoperative eyelid reconstruction or corrective surgery could be a viable option to enhance the outcomes of the two-step LK procedure, helping to prevent epithelial defects.

In addition, we noted the development of peripheral neovascularization, which, although unavoidable, was observed via Figure 1D. At the 8-month follow-up, neovascular growth was confined to the scleral limbus of the anterior lamellar keratolimbal graft from the second step of the surgery, without significantly affecting the posterior lamellar graft with donor endothelial cells from the first step. This containment of neovascularization is considered beneficial in reducing the risk of rejection and corneal melting, ultimately achieving better outcomes compared to traditional PK.

In summary, we report the first case of the Two-Step LK in treating corneal perforation caused by severe alkali burns. The outcomes in preventing graft dissolution and maintaining postoperative corneal function are promising. Future studies should focus on long-term efficacy, safety observations, and expanding indications for this approach.

## Data Availability

The raw data supporting the conclusions of this article will be made available by the authors, without undue reservation.
